# An integrated bioinformatics approach for identification of key modulators and biomarkers involved in atrial fibrillation

**DOI:** 10.34172/jcvtr.025.33347

**Published:** 2025-06-28

**Authors:** Summan Thahiem, Ayesha Ishtiaq, Faisal Iftekhar, Muhammad Ishtiaq Jan, Iram Murtaza

**Affiliations:** ^1^Department of Biochemistry, Faculty of Biological Sciences, Quaid-i-Azam University, Islamabad, Pakistan; ^2^Department of Cardiovascular Surgery, Lady Reading Hospital Peshawar, Peshawar, Pakistan; ^3^Department of Chemistry, Faculty of Chemical & Pharmaceutical Sciences, Kohat University of Science and Technology, Kohat, Pakistan

**Keywords:** Atrial fibrillation (AFib), Bioinformatic approaches, Gene regulatory network, miRNAs, Protein-protein interaction, Sympathetic cardio-renal axis

## Abstract

**Introduction:**

Atrial fibrillation (AFib) is a sustained form of cardiac arrythmia that occurs due to sympathetic overdrive, neurohumoral and electrophysiological changes. Sympatho-renal modulatory approach via miRNA-based therapeutics is likely to be an important treatment option for AFib. The study was aimed to unravel the common miRNAs as therapeutic targets involved in sympatho- renovascular axis to combat AFib.

**Methods:**

We employed the bioinformatics approach to discover differentially expressed genes (DEGs) from microarray gene expression datasets GSE41177 and GSE79768 of AFib patients. Concomitantly, genes associated with sympathetic cardio-renal axis, from Genetic Testing Registry (GTR) of National Center for Biotechnology Information (NCBI) were also analyzed. Overlapping miRNAs that target the maximum number of genes across all three pathological conditions perpetuating AFib were shortlisted. To confirm the reliability of the identified miRNAs, differential expression analysis was performed on miRNA expression profiles GSE190898, GSE68475, GSE70887 and GSE28954 derived from AFib patient samples.

**Results:**

ShinyGO analysis revealed enrichment in beta-adrenergic signaling, calcium signaling, as well as G protein-coupled receptor (GPCR) signaling involved in post synaptic membrane potential. The intersection of top 10 modules in miRNA-mRNA network revealed hub miRNAs having highest node degree, maximum neighborhood component (MNC), and maximal clique centrality (MCC) scores. Differential expression analysis revealed hub miRNAs identified through integrated approach were found to be significantly dysregulated in AFib patients.

**Conclusion:**

This integrated approach identified 6 hub miRNAs, 4 reported (miR-101-3p, miR-23-3p, miR-27-3p, miR-25-3p) and 2 novel (miR-32-5p, miR-92-3p) miRNAs that might act as putative biomarkers for AFib.

## Introduction

 Atrial fibrillation (AFib) is one of the most common forms of arrhythmia, characterized by an irregular and often rapid heartbeat. According to the Global Burden of Disease (GBD) report, the estimated prevalence of AFib has reached 46.3 million.^1^ Its prevalence is expected to increase twice or more in the next 40 years.^2^ AFib is associated with a twofold increase in premature mortality due to adverse cerebrovascular and cardiovascular events.^3^ The etiology of AFib is multifactorial, characterized by wide range of comorbidities ranging from sympathetic overdrive to neurohumoral and hemodynamic pathophysiological mechanisms.^4^

 Sympathetic nervous system (SNS) activation has been well-known as a fundamental determining factor in AFib pathophysiology and has a significant influence on the structural integrity and electrical conductivity of both kidney and heart.^5^ Aberrant SNS activation can induce heterogeneous changes such as excess release of catecholamines in the circulation, hyperactivation of renin–angiotensin–aldosterone system (RAAS), and spontaneous calcium release, enhanced beta-adrenoceptor activity thus elevating arterial hypertension and deteriorating kidney function.^6,7^ These actions contribute to long-term elevated arterial pressure, electro-structural remodeling characterized by conduction abnormalities and increased automaticity.^8^ AFib and renovascular hypertension (RVH) frequently coexist and have a close bidirectional relationship. The incidence of AFib has been widely associated with concomitant destruction of kidney physiology.^9^ The onset of AFib in patients with renal hypertension may reflect mechanical stress in the atrium.^10^ There is increasing recognition of the crucial role of the renin angiotensin aldosterone cascade in the etiology of cardio-renal hypertension, culminating AFib.^11^

 The role of epigenetic modifications in the pathogenesis of AFib has been documented in a variety of studies, among which miRNAs have acquired significant importance in modulating cardiovascular function.^12,13^ The accurate diagnosis of disease-vulnerable individuals is of paramount importance. For that the identification of miRNAs and their association with disease phenotype for both prognostic and diagnostic purposes is currently under the limelight of research.

 miRNAs are single-stranded RNAs that are non-coding and nearly 22 nucleotides in length. They function as “fine tuners” of gene expression and regulation. Due to their phylogenetic species conservation, highly consistent and stable expression profile, and simplicity of detection, they are regarded as best reporters of disease phenotypes. In this context, computational approaches have been an invaluable tool for miRNA expression profiling, which may aid in the early detection of chronic diseases. ^14,15^

 Interplay of miRNAs in autonomic cross talk between kidney and heart is the best choice for exploring the pathogenesis of AFib. To the best of our knowledge, no systematic research-based study has been performed to identify highly associated genes and miRNAs of AFib using an integrated multi-step bioinformatics analytic pipeline. Sympatho-renal modulatory approach via miRNA-based therapeutics is likely to be an important treatment option for AFib. Prior studies were limited to identification hub genes targeting AFib alone by analyzing different datasets,^16-19^ however, no previous study has employed combinational approach by targeting genes underlying the pathological sympathetic cardio-renal axis that helps in identification of highly potent miRNAs. These miRNAs not only regulate genes involved in AFib but also influence genes that contribute to AFib development risk.

**Figure 1:**
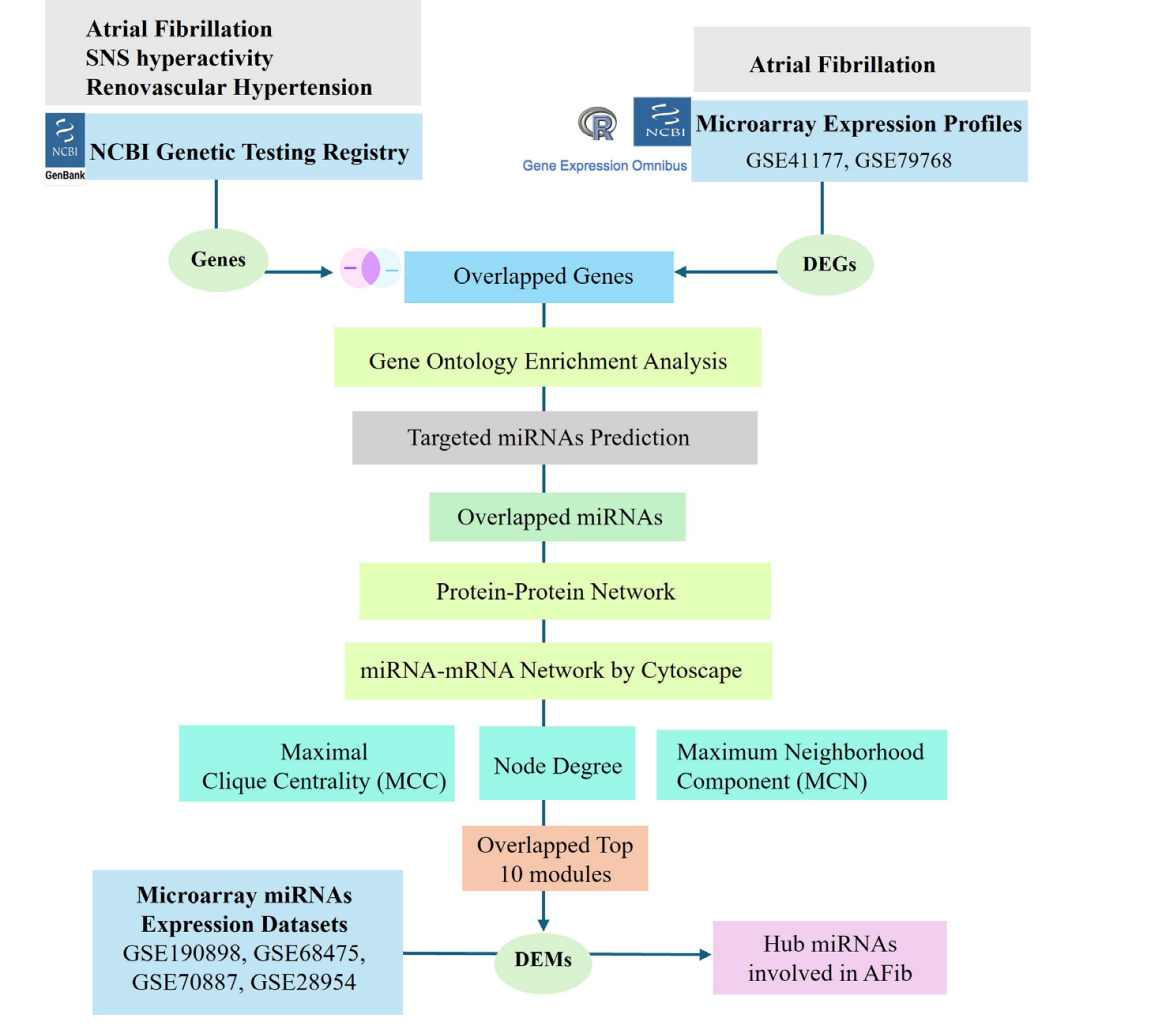
The workflow of integrated analysis used in current study

 The complex interplay of heart failure and chronic kidney disease in atrial fibrillation creates a mutually reinforcing cycle that escalate disease development. ^20-22^ Therefore, the aim of study was to identify potential hub miRNAs by integrating previously published microarray datasets of both genes and miRNAs of AFib patients. The workflow of hub genes and miRNA screening curation pipeline from data acquisition, meta-analysis, identification of differentially expressed entities, to downstream network analysis is shown in Figure 1.

## Materials and Method

###  Acquisition of disease-associated genes 

 In this study, we analyzed the critical pathophysiological factors contributing to atrial fibrillation, especially focusing on brain-kidney-heart axis spanning from sympathetic nervous system neurohormones to renal hypertension. Thus, with the help of bioinformatics repositories NCBI GTR and Online Mendelian Inheritance in Man (OMIM), we selected highly specific disease linked genes that have been investigated clinically for that specific pathological condition.^23^

###  Selection of microarray dataset for comprehensive screening of genes (the discovery cohort)

 Microarray expression profiles related to AFib were screened and curated from GEO repository (https://www.ncbi.nlm.nih.gov/geo/).^24^ We omitted studies involving drug treatments or any other interventions, cell lines, transfected or transgenic tissues. Microarray datasets passing these stringent criteria were selected. For discovery cohort, datasets GSE41177 and GSE79768 of atrial fibrillation patients were retrieved (Table 1). After normalization and annotation of series matrix files, expression data were then analyzed through Affy package and Limma Package in R software. The Limma package specifies functions such as lmFit, eBayes, and topTable for linear modeling along with statistical testing.

**Table 1:** Summary of gene expression datasets of AFib patients

**Geo Accession ID**	**Title**	**Platform**	**Sample Size**
GSE41177	Region-specific gene expression profiles in left atria of patients with valvular atrial fibrillation	Affymetrix Human Genome U133 Plus 2.0 Array	38
GSE79768	Atrial fibrillation is associated with altered left-to-right atria gene expression ratio: implications for arrhythmogenesis and thrombogenesis	GPL570[HG-U133_Plus_2] Affymetrix Human Genome U133 Plus 2.0 Array	26

###  Data processing, screening and identification of differentially expressed genes

 The raw data of microarray expression profile of patients and control were preprocessed (background correction, annotation, gene symbol transformation, and normalization) to reduce confounding effects. The probes with no annotation information were deleted. Then, Bioconductor package limma was used to identify the differentially expressed genes (DEGs). Screening parameter for identification of DEGs was based on adjusted *P* value < 0.05, |log2 fold change| > 1 and -log10 (*P* value) > 1.3. Additionally, a volcano plot was generated to visualize significant upregulated and downregulated entities.

###  Screening and visualization of hub genes

 Top ranked hub genes were deployed that were common in GTR and DEGs from microarray datasets. For data visualization, we used AI based Tableau software (https://www.tableau.com/). Overlapping entities among DEGs and Genes list obtained from NCBI GTR and OMIM were visualized using Venn diagram, utilizing the online tool InteractiVenn (https://www.interactivenn.net).^25^

###  Gene set functional enrichment analysis

 The gene enrichment analysis was executed for genes associated with Sympathetic Cardio-Renal Axis, to identify statistically significant signaling pathways using ShinyGO 0.80 (http://bioinformatics.sdstate.edu/go/). A screening threshold of false discovery rate (FDR) < 0.05 and *P*< 0.01 was applied for statistical significance of data.^26^

###  miRNAs target prediction

 Hub genes were than integrated for miRNA target prediction using open-source platforms; TargetScan database (http://www.targetscan.org), and miRbase (http://mirtarbase.mbc.nctu.edu.tw) for studying the miRNA-mRNA interaction.^27,28,29^ The selection criteria for miRNA was based on thermodynamic stability, evolutionary conservation, seed matching, and site accessibility to improve the target prediction fidelity. Only 8-mer, (human, rat, and mouse) species conserved miRNAs were selected for each gene.

###  Protein-Protein Interaction (PPI) network construction

 The Search Tool for the Retrieval of Interacting Genes (STRING) database Version 12.0 (https://string-db.org/)^30^ was used to predict and track PPI interactions. The analysis of interactions between different proteins provides new insight to study the pathophysiological mechanisms of AFib. Using DisGeNET algorithm of Metascape (https://metascape.org) ^31^ genes were further examined for functional enrichment analysis.

###  miRNA-mRNA network construction and hub modules identification

 Cytoscape (v3.10.1) Plugin CytoHubba ^32^ was employed to construct miRNA-mRNA network to identify densely connected entities based on 3 topologies; node degree, maximal clique centrality (MCC), and maximum neighborhood component (MNC). Entities overlapping across these topologies were selected as hub miRNAs and hub genes.

###  Selection of miRNA microarray datasets for validation of hub miRNAs (the validation cohort)

 For validation cohort, the miRNA profiling datasets GSE190898, GSE68475, GSE70887 and GSE28954 of atrial fibrillation patients were retrieved (Table 2). After normalization and annotation of series matrix files, expression data were then analyzed through affy package and Limma Package in R software.

**Table 2:** Summary of miRNA expression datasets used in this study

**Accession ID**	**Tittle**	**Platform**	**Samples**
GSE190898	Atrial Fibrillation Patient's Urine miRNA Microarray	GPL21572 [miRNA-4] Affymetrix Multispecies miRNA-4 Array	8
GSE68475	Characterizing the global changes in miRNA expression in human atrial appendages with persistent atrial fibrillation.	GPL15018 Agilent-031181 Unrestricted_Human_miRNA_V16.0_Microarray 030840 (Feature Number version)	21
GSE70887	Microarray analysis of miRNAs in atrial tissue from chronic AF patients	GPL19546 Agilent-021827 Human miRNA Microarray [miRBase release 17.0 miRNA ID version]	8
GSE28954	Valvular heart disease and atrial fibrillation regulate microRNA expression profiles in left and right atria differently	GPL10850 Agilent-021827 Human miRNA Microarray (V3) (miRBase release 12.0 miRNA ID version)	34

## Results

###  Screening and identification of common genes among genes of cardio-renal axis and AFib discovery dataset

**Figure 2:**
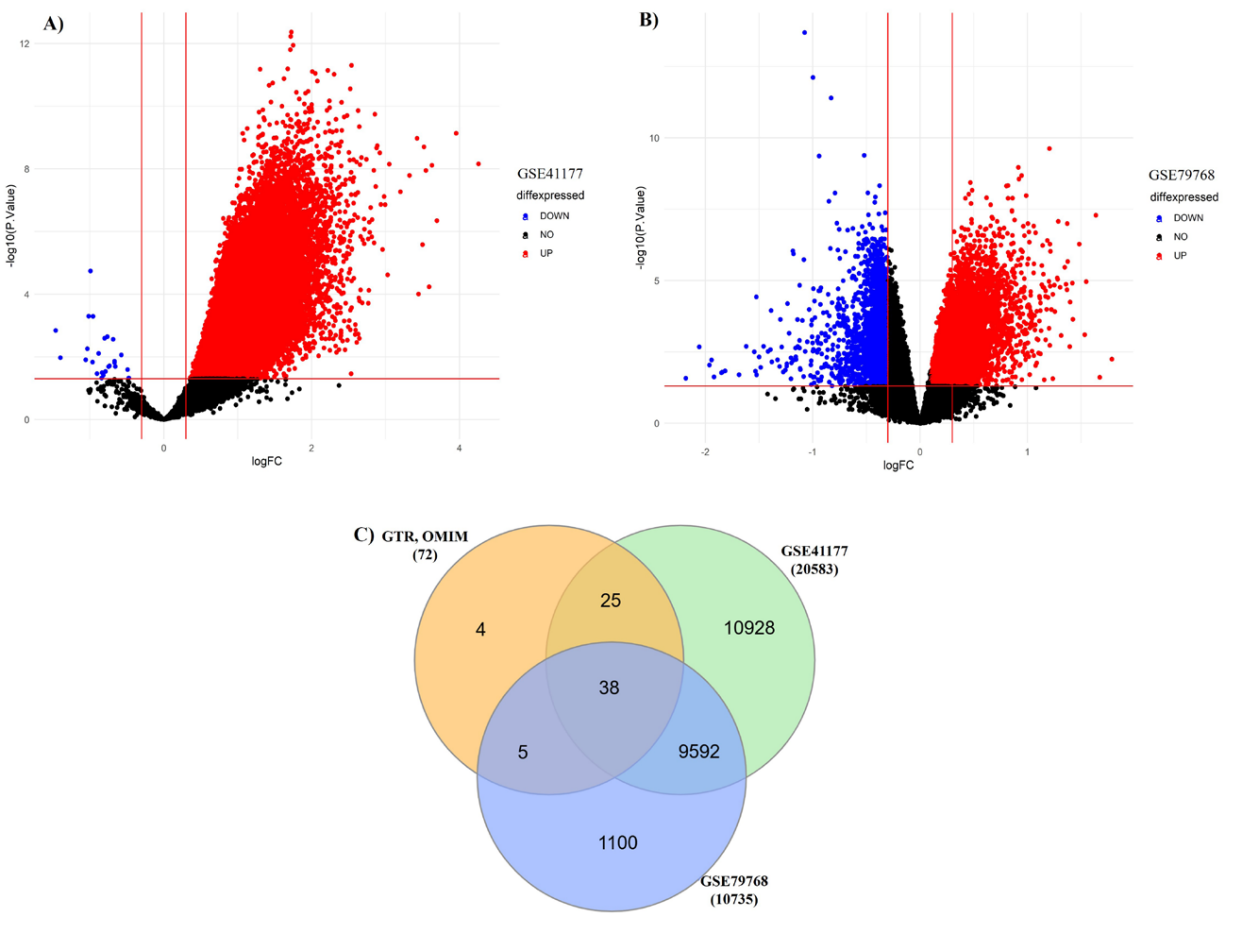
Volcano plots of DEGs; illustrating the distribution of differentially expressed genes according to the logFC and -log(*p*-value) values. **(A)** GSE41177** (B) **GSE79768 **(C) **Venn diagram of overlapping DEGs

**Figure 3:**
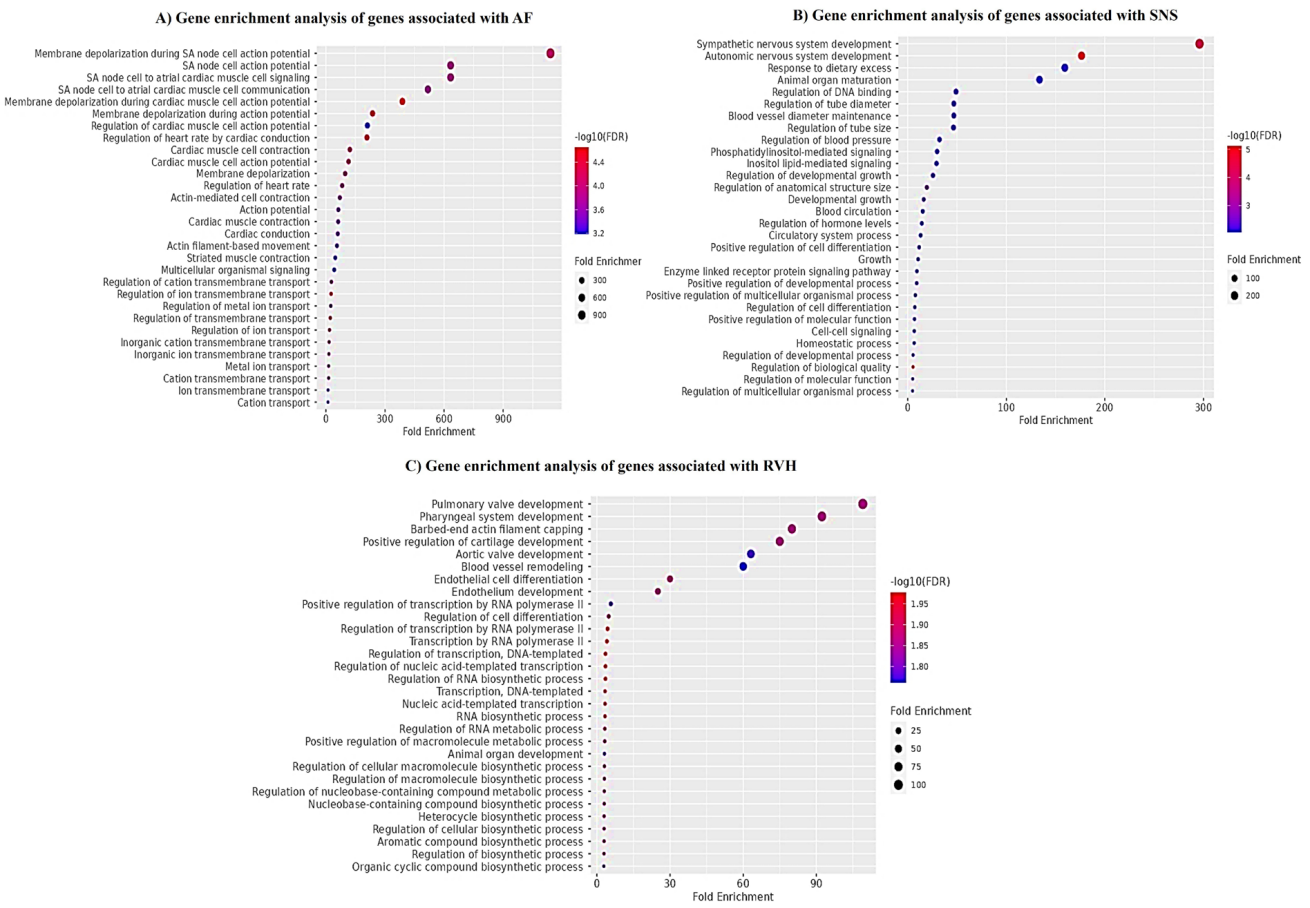
Gene enrichment analysis** (A) **AFib associated genes. Many of these genes are known to underpin molecular responses such as membrane depolarization, sinoatrial action potential generation, muscles contraction, regulation of ions transmission and regulation of heart rate. **(B)** SNS hyperactivity associated genes. Enriched terms include sympathetic nervous system development, autonomic nervous system activity, circulatory system processes, cell-cell signaling, homeostatic signaling. **(C)** RVH associated genes. Genes showed enrichment in processes including blood vessels remodeling, pulmonary and aortic valves development and pharyngeal system development

 We selected disease specific genes from bioinformatics repositories e.g., NCBI GTR and OMIM as given in Table S1. Moreover, microarray gene expression profiles GSE41177 and GSE79768 of AFib patients and controls were also analyzed. The DEGs are represented in volcano plots according to the logFC and -log(*P*-value) values as shown in (Figure 2A & B) (Table S2 & S3). We compared gene lists associated with AFib derived from bioinformatics repositories and DEGs from microarray gene expression datasets. 38 overlapping genes were found to be involved in Sympathetic Cardio-Renal Axis and AFib. The overlapping genes are represented in Venn diagram (Figure 2C). For in-depth analysis of gene sets, enrichment analysis was performed separately on all three genes sets associated with AFib, SNS activity and RVH as shown in Figure 3.

###  miRNA profiling of overlapped genes associated with Sympathetic Cardio-Renal Axis

**Figure 4:**
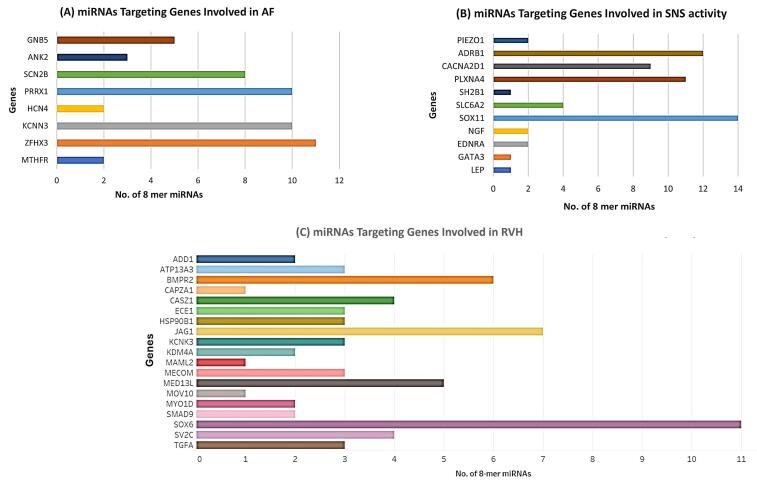
Graphical representation of miRNAs associated with Sympathetic Cardio-Renal Axis** (A)** miRNAs targeting genes associated with AFib.** (B)** miRNAs targeting genes associated with SNS activity. **(C)** miRNAs targeting genes associated with RVH. X axis depicts the number of miRNAs whereas, Y axis shows the list of respective genes having 8-mer binding sites

 Only species conserved miRNAs having 8-mer binding seed region in the 3’ UTR of genes involved in the progression of AFib, SNS activity and RVH have been shortlisted for downstream analysis. The data are given in Table S4. Graphical representation of these genes with their respective 8-mer miRNAs are shown in Figure 4.

###  Commonly expressed miRNAs and targeted genes involved in Sympathetic Cardio-Renal Axis

**Figure 5:**
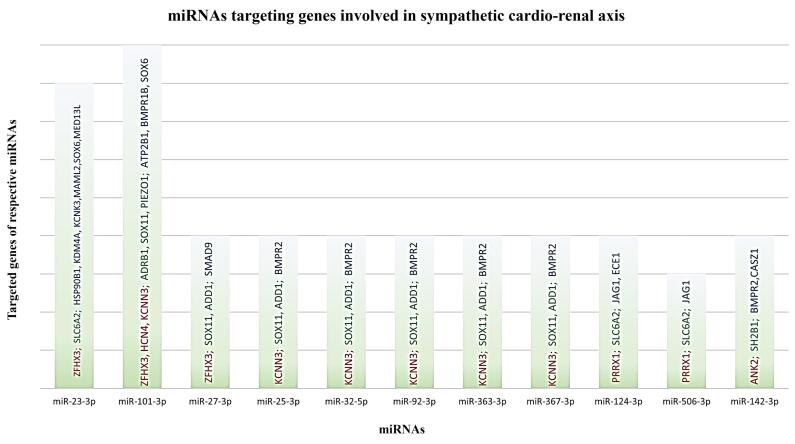
Graphical representation of common miRNAs involved in all three pathologies A single miRNA can target more than one gene and is involved in more than one pathophysiological mechanism. The graph shows 11 miRNAs (miR-23-3p, miR-101-3p, miR-27-3p, miR-25-3p, miR-32-5p, miR-92-3p, miR-363-3p, miR-367-3p, miR-124-3p, miR-506-3p, miR-142-3p) that can target common genes involved in sympatho-excitation in AFib pathophysiology. Red color represents AFib linked genes; green color represents genes regulating SNS activity whereas blue depicts genes involved in RVH respectively

 RVH and AFib co-exist frequently, and share common putative mechanisms, suggesting that common pathophysiological processes may drive both pathologies. ^33^ Hence manipulating the expression of genes involved in both pathologies can ameliorate the disease phenotype at earliest. Subsequently, for this purpose, we shortlist hub miRNAs common in all pathologies that target more than one gene. The key finding of our data confirms that 11 miRNAs (miR-23-3p, miR-101-3p, miR-27-3p, miR-25-3p, miR-32-5p, miR-92-3p, miR-363-3p, miR-367-3p, miR-124-3p, miR-506-3p, miR-142-3p) are targeting common genes involved in the development and progression of cardiac arrythmia and AFib as shown in Figure 5.

###  Gene enrichment analysis for genes associated with Sympathetic Cardio-Renal Axis

**Figure 6:**
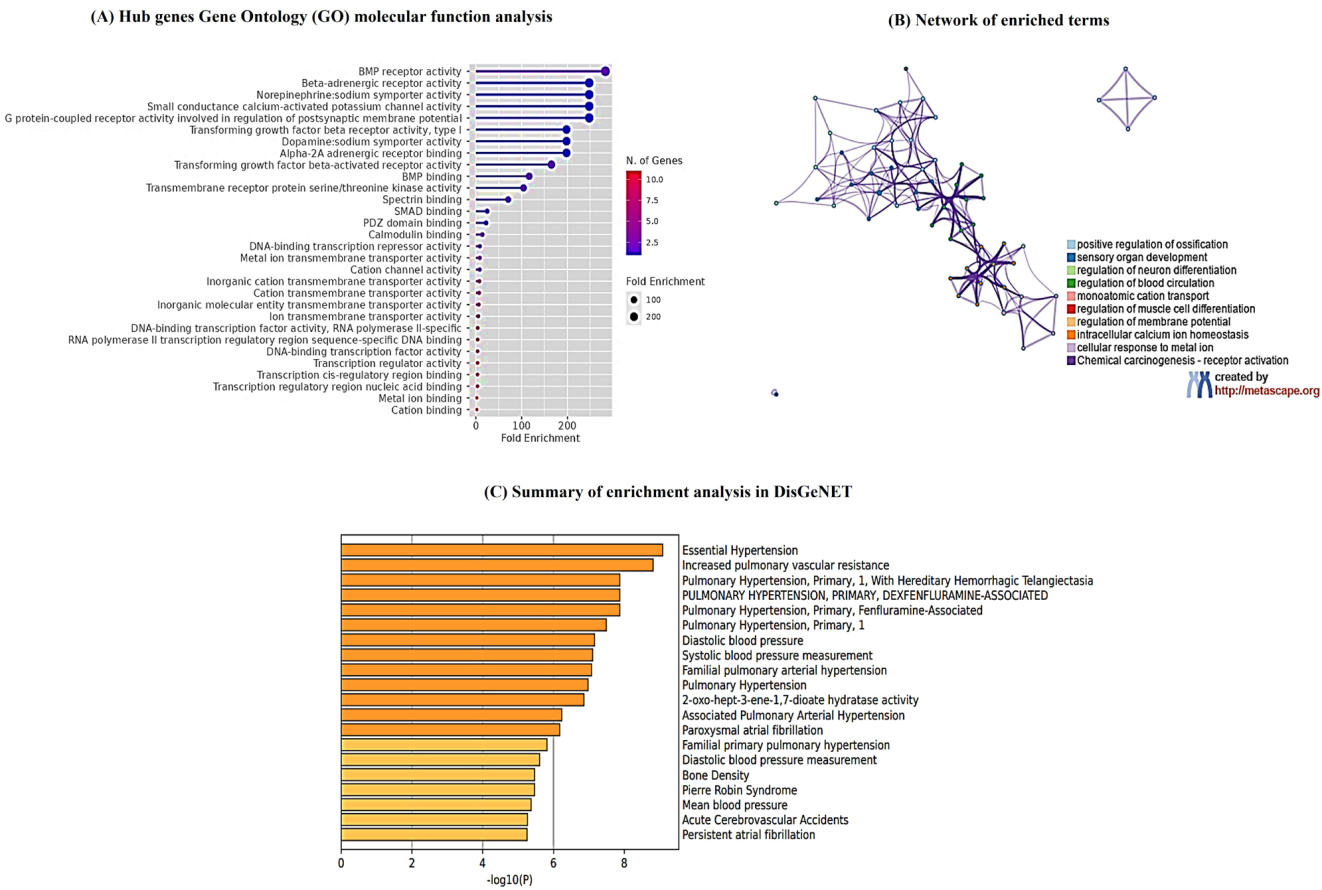
Gene enrichment analysis of differentially expressed genes involved AFib pathogenesis **(A)** ShinyGO gene enrichment analysis revealed DEGs involvement in beta-adrenergic signaling, calcium signaling, non-epinephrine sodium symporter activity as well as GPCR signaling involved in post synaptic membrane potential. The x-axis represents the fold enrichment count, and the y-axis depicts enriched pathways. The color gradient from red to blue indicates the number of genes and concomitant fold enrichment variation in decreasing order. **(B)** Network of enriched terms by Metascape. Here each circle node represents a specific node, and size is proportional to the number of genes of that cluster, whereas color represents its cluster identity. Terms having similarity index > 0.3 are linked by an edge whose thickness represents the similarity score. **(C)** DisGeNET database enrichment analysis of different pathological conditions associated with genes. X axis represents -log10(P) value whereas y axis represents highly associated diseases with DEGs such as hypertension, vascular resistance, blood pressure, atrial fibrillation

 To investigate the specific biological function classification of DEGs and to validate our shortlisted genes, we executed the gene enrichment analysis using ShinyGO database. These genes showed enrichment in beta-adrenergic signaling, calcium signaling, non-epinephrine sodium symporter activity as well as GPCR signaling involved in post synaptic membrane potential as shown in Figure 6A. To further elucidate the relationship between DEGs, a network plot was constructed which displays enrichment in blood circulation, regulation of membrane potential and calcium ion homeostasis as shown in Figure 6B. Moreover, using DisGeNET algorithm, disease enrichment analysis demonstrated that hub genes were significantly associated with hypertension, vascular resistance, blood pressure, and atrial fibrillation as shown in Figure 6C.

###  Construction of the protein-protein interaction (PPI) network and analysis of significant modules

**Figure 7:**
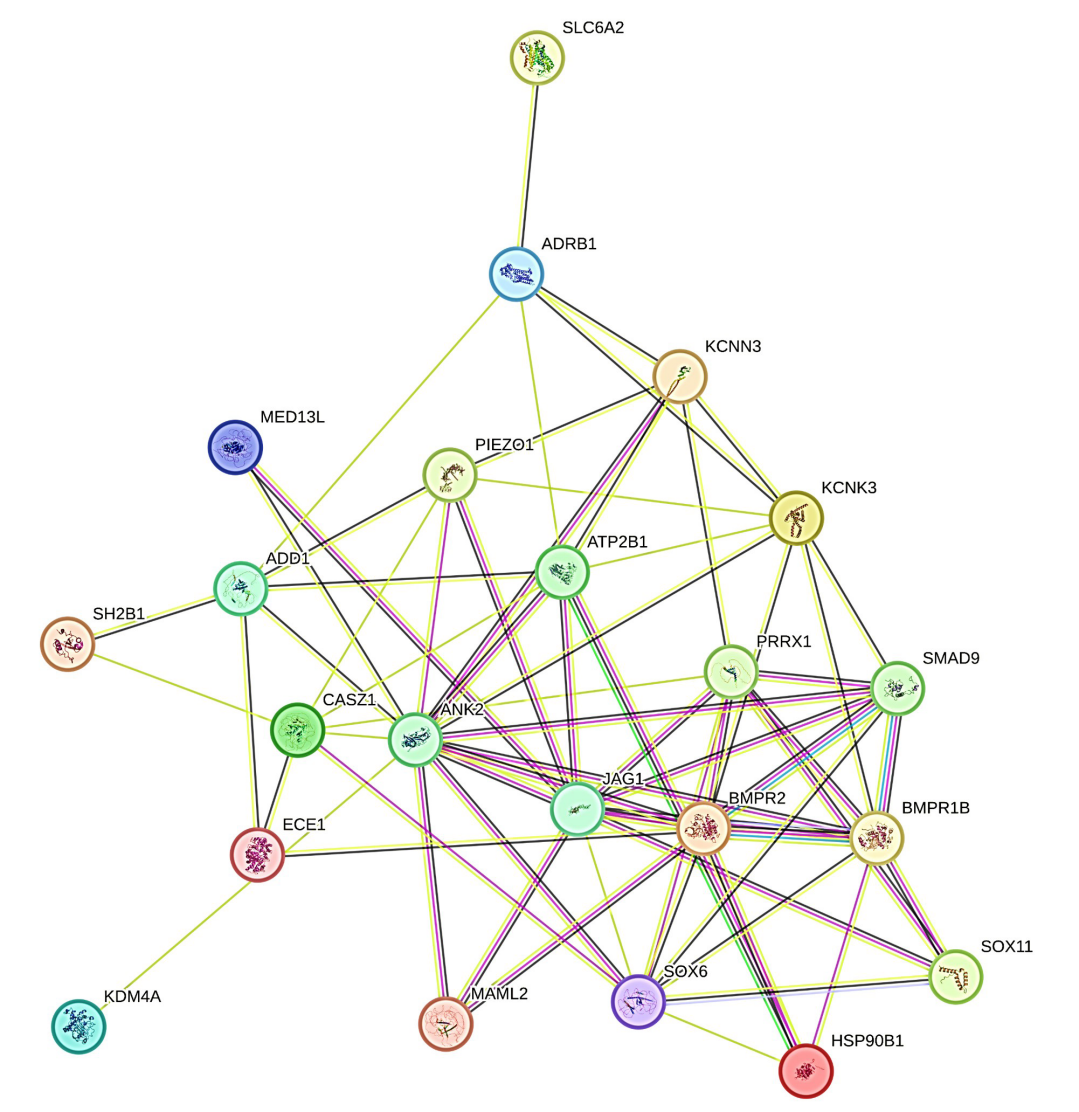
Protein–protein interaction (PPI) network; 22 genes were found to be predominantly involved in the PPI network, in which (i-e., ANK2, JAG1, BMPR2, BMPR1B, SOX6, ATP2B1, KCNK3, PRRX1, CASZ1, SMAD9) exhibit highest node degree interactions with *p*-value of 1.11e-16

 The precise PPI network was constructed with a high-confidence interaction score (> 0.7) on the online tool STRING. 22 genes were found to be involved in the PPI pairs as shown in Figure 7. In this PPI network, there were 67 edges and 22 nodes having a PPI enrichment *p*-value of 1.11e-16. We selected 10 hub genes (i-e., ANK2, JAG1, BMPR2, BMPR1B, SOX6, ATP2B1, KCNK3, PRRX1, CASZ1, SMAD9) having the highest node degree interactions in the gene regulatory network as shown in (Table 3).

**Table 3:** Hub nodes, having the highest degree interactions in the gene regulatory network

**Nodes**	**Degree Score**	**Nodes**	**Degree Score**
ANK2	14	PRRX1	8
JAG1	11	SMAD9	7
BMPR2	10	ADD1	6
BMPR1B	9	KCNK3	8
SOX6	9	PRRX1	8
ATP2B1	8	KCNN3	6
KCNK3	8	PIEZO1	6

###  mRNA-miRNA network analysis 

**Figure 8:**
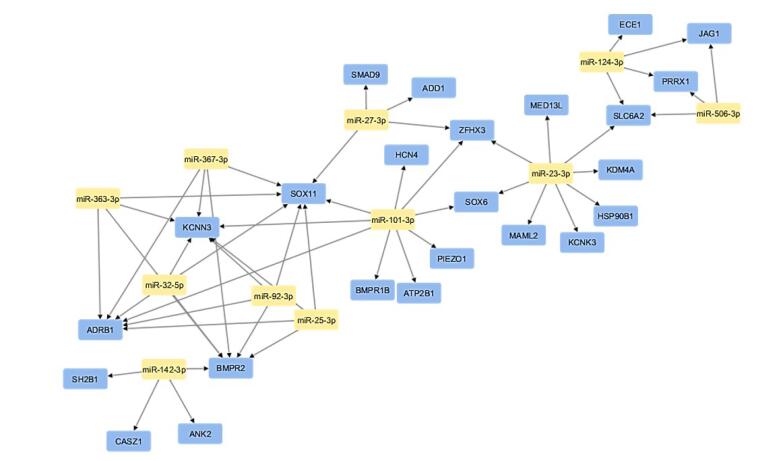
miRNA-mRNA network using Cytoscape;Yellow color nodes represent miRNAs whereas blue color nodes represent their target genes

**Figure 9:**
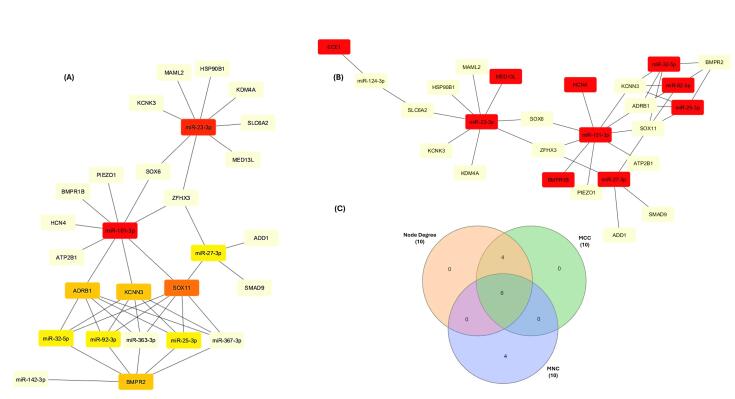
Identification of the hub genes and miRNAs **(A)** Top ranked genes and miRNAs using maximal clique centrality (MCC) algorithm and Node Degree rank. **(B)** Top ranked modules identified according to maximum neighborhood component (MNC) method. **(C)** Overlapped top ranked miRNAs and mRNAs from the MNC, MCC, and node degree methods. The red color nodes represent genes with highest sores, whereas the yellow color node represents low score

 mRNA-miRNA network was constructed using Cytoscape (Figure 8). CytoHubba plugin identified crucial network hubs through three distinct methods: the maximal clique centrality (MCC) score, maximum neighborhood component (MNC) score, and highest degree score (Figure 9A & B). The intersection of top 10 modules having highest node degree, MNC, and MCC scores (Table 4) were considered as hub miRNAs, as shown in Venn diagram (Figure 9C). This robust pipeline for prioritizing potential disease modulators identified 6 hub miRNAs (miR-101-3p, miR-23-3p, miR-27-3p, miR-25-3p, miR-32-5p, miR-92-3p).

**Table 4:** Top 10 genes and miRNAs scores ranked by MCC, MNC and Degree method

**Rank**	**Name**	**Degree Score**	**Rank**	**Name**	**MCC Score**	**Rank**	**Name**	**Closeness Score**	**Rank**	**Name**	**MNC Score**
1	miR-101-3p	9	1	miR-101-3p	9	1	miR-101-3p	17.45	1	MED13L	1
2	miR-23-3p	8	2	miR-23-3p	8	2	SOX11	16.316667	1	miR-92-3p	1
3	SOX11	7	3	SOX11	7	3	miR-23-3p	16.045238	1	miR-32-5p	1
4	KCNN3	6	4	KCNN3	6	4	KCNN3	15.15	1	HCN4	1
4	ADRB1	6	4	ADRB1	6	4	ADRB1	15.15	1	miR-25-3p	1
4	BMPR2	6	4	BMPR2	6	6	ZFHX3	15.033333	1	BMPR1B	1
7	miR-27-3p	4	7	miR-27-3p	4	7	miR-27-3p	14.116667	1	miR-27-3p	1
7	miR-25-3p	4	7	miR-25-3p	4	8	SOX6	13.866667	1	ECE1	1
7	miR-32-5p	4	7	miR-32-5p	4	9	BMPR2	13.527381	1	miR-101-3p	1
7	miR-92-3p	4	7	miR-92-3p	4	10	miR-25-3p	13.378571	1	miR-23-3p	1

###  Screening of differentially expressed miRNAs (DEMs) in clinical AFib patient’s datasets

**Figure 10:**
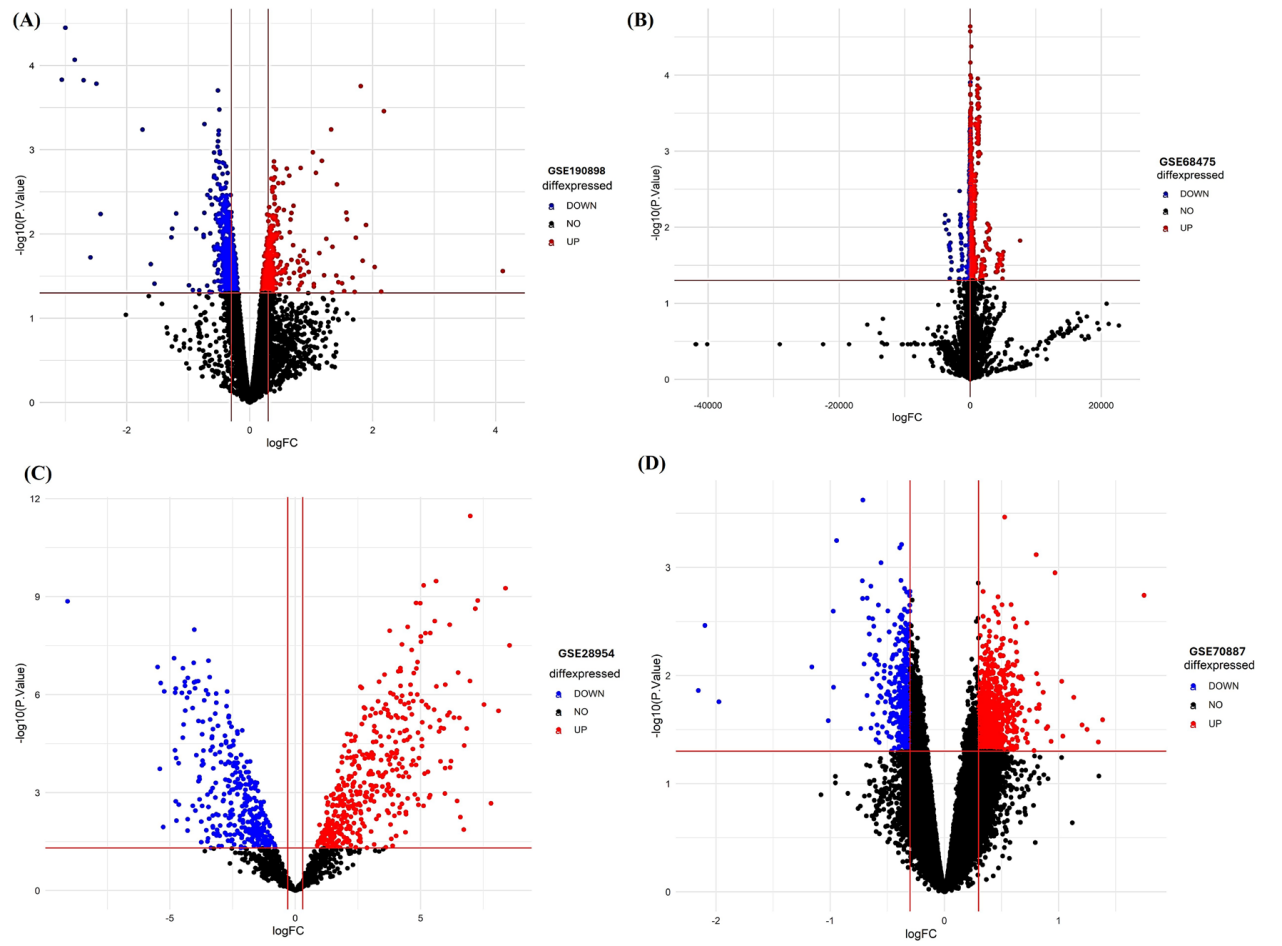
Volcano plots of differentially expressed miRNAs from multiple datasets **(A)** GSE190898 **(B)** GSE68475, **(C)** GSE28954 **(D) **GSE70887

 We then sought to determine the reproducibility of the initial profiling of hub miRNA signatures in multiple independent datasets (Table 5). DEMs passing the criteria of *P*< 0.05 and log_2_|fold change| > 1 were considered as the potential candidates, as given in (Table S5-S8). Differentially expressed hub miRNAs are shown in Volcano plots in Figure 10 A-D.

**Table 5:** Differentially expressed hub miRNAs

**miRNAs**	**log FC**	***p*** **-value**	**Datasets Accession IDs**
**hsa-miR-101**	8.975409	0.04108	GSE68475
	1.498829	0.0384	GSE190898
**hsa-miR-23a**	6.814861	0.042828	GSE68475
**hsa-miR-25**	-9.20899	0.005195	GSE68475
	0.241439	0.03505	GSE28954
	-0.13165	0.043473	GSE70887
**hsa-miR-32**	9.508575	0.006066	GSE68475
**hsa-miR-92b**	11.60204	0.004529	GSE68475
**hsa-miR-27**	15.41865	0.00703	GSE68475

## Discussion

 The vicious cycle of three systemic biological systems, i.e., sympathetic, renovascular, and cardiovascular systems, form a most crucial and responsive network throughout the body that is responsible for AFib. ^34,35^ Thus, it is of paramount importance to scrutinize the molecular mechanisms that exacerbate disease severity in AFib patients. For this, an attempt has been made to find out the hub miRNAs through integrated bioinformatics approaches.

 The genes from GWAS enlisted in NCBI GTR, OMIM, and Gene Card and differentially expressed genes of GSE41177 and GSE79768, only overlapped genes were selected for further analysis. For in-depth analysis of gene sets, ShinyGO enrichment analysis was performed separately on all three genes sets. Taken together, downstream analysis of hub genes and associated miRNAs with AFib and symaptho-cardiorenal axis render valuable information regarding crucial aspects of affected biological processes, dysregulated cellular pathways, associated diseases, and diagnostic biomarkers. Many of the genes associated with AFib were found to underpin molecular responses such as membrane depolarization, sinoatrial action potential generation, and muscles contraction, regulation of ions transmission and regulation of heart rate. Whereas analysis of genes associated with SNS activity exhibited enriched terms in sympathetic nervous system development, autonomic nervous system activity, circulatory system processes, cell-cell signaling, and homeostatic signaling. Enrichment analysis of RVH associated genes showed enrichment in processes including blood vessels remodeling, pulmonary and aortic valves development and pharyngeal system development. Next, we performed computational screening of highly specific and accurate miRNAs interacting with shortlisted genes by using TargetScan database. MiRNAs having 8-mer binding seed region in the 3ÚTR of genes were selected. We shortlisted miRNAs (miR-23-3p, miR-101-3p, miR-27-3p, miR-25-3p, miR-32-5p, miR-92-3p, miR-363-3p, miR-367-3p, miR-124-3p, miR-506-3p, miR-142-3p) common in all pathologies (AFib, SNS hyperactivity and RVH).

 Target genes were then subjected to DisGeNET database for enrichment analysis with respect to diseases. Results revealed enrichment in hypertension, vascular resistance, blood pressure, atrial fibrillation which further endorsed our findings. The precise PPI network was constructed on STRING. 10 hub genes (i-e., ANK2, JAG1, BMPR2, BMPR1B, SOX6, ATP2B1, KCNK3, PRRX1, CASZ1, SMAD9) were found to have the highest node degree interactions in the gene regulatory network. Furthermore, using CytoHubba plug-in, we identified top level modules using 3 different methods i.e., the maximal clique centrality (MCC), maximum neighborhood component (MNC), and degree method. The intersection of top 10 modules having highest node degree, MNC, and MCC scores were considered as hub miRNAs (i.e., miR-101-3p, miR-23-3p, miR-27-3p, miR-25-3p, miR-32-5p, miR-92-3p). We integrated miRNA expression profiles of AF samples from 4 GEO datasets and analyzed the data using R software and bioinformatics tools. The expression of hub miRNAs was also found to be significantly dysregulated in patients as compared to healthy individuals.

 After comprehensive profiling of miRNAs, miR-101-3p was found to be key miRNA in co-expression network having highest MCC score. MiR-101-3p has protective effects on atrial fibrillation as it increases the atrial effective refractory period, thus reducing the AFib incidence. ^36^ Another study also reported that miR-101 could inhibit fibrosis by targeting proto-oncogene (c-Fos), a potential biomarker for neuronal activity and transforming growth factor-β1 (TGF-β) coding gene. MiRNA target prediction through databases revealed ZFHX3, HCN4, KCNN3, ADRB1, SOX11, PIEZO1, ATP2B1, BMPR1B and SOX6 contain 8-mer binding sites for miR-101-3p. However, no *in vivo* experimentation regarding aforementioned genes with miR-101-3p has been reported yet.

 Apart from electrical remodeling due to ionic imbalance, structural remodeling is another crucial process in AFib pathophysiology and is marked by atrial fibrosis. ^37^ Our findings also emphasize miR-27-3p as the most important regulator of AFib pathogenesis. *In silico* analysis for miRNA prediction using TargetScan database showed that ZFHX3, SOX11, ADD1 and SMAD9 are potential target genes of miR‐27‐3p. Previous study also reported that miR‐27‐3p promote atrial fibrosis by targeting SMAD signaling pathway thus leading to atrial fibrosis.^38^ The pathophysiology of AFib is manifested by upregulation of fibrosis related genes and down regulation of ion channels encoding genes that cause disturbance in cardiac electrical activity. miR-27-3p also reportedly involved in electrical remodeling through HOXa10, resulting in a decreased expression of voltage-gated sodium (Nav1.5), potassium (Kv4.2), and calcium (Cav1.2) channels encoded by SCN5A, KCND2 and CACNA1C gene respectively. ^39^ Concomitantly another study reported that miR-27b increases vulnerability to cardiac arrhythmia leading to conduction disturbance by targeting atrial gap junction coding gene connexin-40 (CX40).^40^

 Predictive analysis by TargetScan database revealed the presence of binding sites of miR-23-3p in 3’ UTR region of genes i.e., ZFHX3, SLC6A2, HSP90B1, KDM4A, KCNK3, MAML2, SOX6 and MED13L. MiR-23-3p is involved in ferroptosis in atrial fibrillation patients by targeting SLC7A11.^41^ Another study reported that miR-23-3p perpetuate AFib progression by regulating TGF-β1.^42^ Similar pattern observed with miR-25-3p that promotes atrial fibrosis by suppressing Dickkopf 3 (Dkk3), an enhancer of SMAD7 expression, that activate SMAD3 and fibrosis-related genes expression.^43^

 Another key finding of our integrated bioinformatic analysis underlies the identification of miR-32-5p and miR-92-5p that has not been reported in prior studies related to AFib. Furthermore, both miRNAs have been computationally predicted to have evolutionary conservation and 8-mer binding sites for calcium-activated potassium channel gene (KCNN3), SRY-box transcription factor (SOX11), Adrenoceptor Beta 1 (ADRB1), and Bone morphogenetic protein receptor type II (BMPR2). MiR-32-5p and miR-92-5p have pleiotropic effects and can target both structural and electrical remodeling causing genes simultaneously, thus highlighting their potential as promising therapeutic targets for the treatment of AFib patients.

 The current study has a few limitations. The hub genes were identified by bioinformatics analysis, necessitating experimental research for validation of the findings. Despite these constraints, this study possesses promise as it offers novel insights into the pathophysiology and therapeutic targets of AFib by employing an integrated multi-step computational workflow to extract high-level evidence from various datasets, hence increasing the utility of available bioinformatics resources.

## Conclusion

 Our study integrated microarray-based miRNA and mRNA expression profiles from datasets comprising multiple cohorts and series of bioinformatics analyses. Concomitantly, disease targets related to sympathetic cardio-renal axis were screened from NCBI GTR and OMIM, which provides rationale in exploring the molecular basis of evading genes and miRNAs. Through extensive bioinformatics analytic workflow, we identified 6 hub miRNAs, 4 previously reported (miR-101-3p, miR-23-3p, miR-27-3p, miR-25-3p) and 2 novel (miR-32-5p, miR-92-3p) miRNAs for AFib that might act as putative biomarkers. The findings of our study are essential for the establishment of precision medicine initiatives, as they contribute to the comprehension of the genetic and molecular basis of AFib. Subsequent research is necessary to substantiate the molecular mechanisms that underline these findings.

## Competing Interests

 The authors declare that they have no conflicts of interest.

## Ethical Approval

 This article does not contain any research involving animals or humans as subjects of research.

## Supplementary Files

Supplementary file Contains Table S1-S8.
